# Deep-Learning-Enhanced TopoStats for the Automated Quantification of DNA and Complex Biomolecular Structures

**DOI:** 10.34133/csbj.0227

**Published:** 2026-09-18

**Authors:** Sylvia Whittle, Tobias A. Firth, Max C. Gamill, Laura Wiggins, Neil Shephard, Toby Allwood, Daniel Brady, Thomas E. Catley, Alice L. B. Pyne

**Affiliations:** ^1^ School of Chemical, Materials and Biological Engineering, University of Sheffield, Sheffield, UK.; ^2^ School of Medicine and Population Health, University of Sheffield, Sheffield, UK.; ^3^ Department of Computer Science, University of Sheffield, Sheffield, UK.

## Abstract

Atomic force microscopy (AFM) enables nanometer-scale, label-free imaging of biomolecules and surfaces under near-native conditions, yet quantitative analysis of AFM data remains limited. This limitation largely arises from the absence of open, automated tools capable of addressing AFM-specific artifacts, data formats, and topographical outputs. Here, we present a substantial advancement to TopoStats, an open-source Python package for AFM image analysis, which introduces advanced analysis algorithms and deep learning capabilities to support more complex samples and richer molecular characterization. The new pipeline facilitates a shift from simple object segmentation and morphometric feature extraction toward automated tracing of molecules through complex crossings, enabling topological classification and curvature analysis. We demonstrate this by differentiating between plasmids with both different topology and sequence using quantitative descriptors, such as the number of crossings and median curvature, that distinguish the samples with statistical significance. Designed for accessibility and reproducibility, TopoStats adheres to the Findable, Accessible, Interoperable, and Reusable (FAIR) for Research Software principles and provides configurable workflows to allow the quantification of subtle structural changes within heterogeneous biological samples. This advances AFM from a fundamentally qualitative visualization technique toward a quantitative analytical tool, revealing properties not accessible with other structural biology techniques.

## Graphical Abstract

**Figure:**
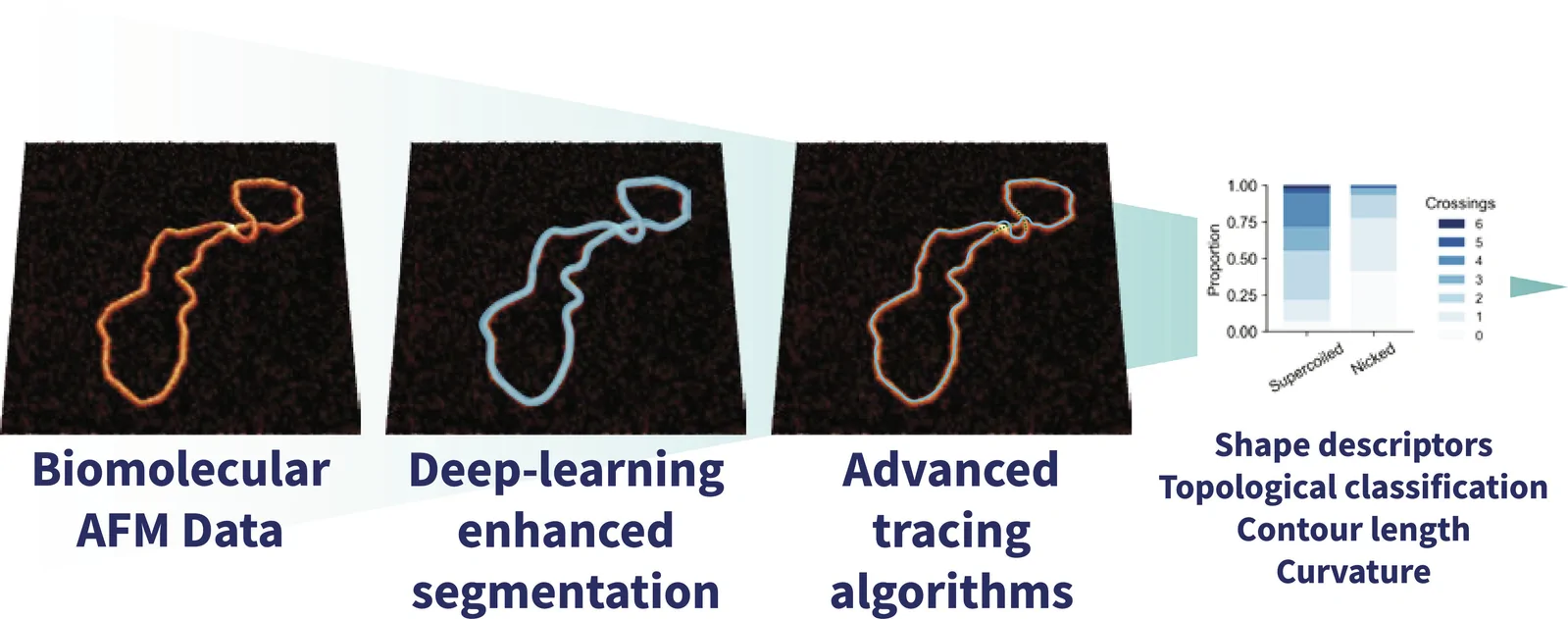

## Introduction

Advances in atomic force microscopy (AFM) technology have made routine high-resolution imaging of biomolecules and polymers accessible to the wider biophysics community. Commercial systems with a stable, high signal-to-noise ratio provide users with the ability to image with subnanometer lateral and picometer axial resolution, allowing for label-free imaging under near-native conditions. The introduction of “tapping mode” allowed for the first image of DNA to be resolved by AFM [1,2], with later technical developments allowing resolution of the major/minor grooves to be readily attainable [3,4]. Similarly, AFM has been used to characterize the structure of pathological protein fibrils associated with Alzheimer’s disease and the heterogeneity of actin filament substructure demonstrating AFM as a viable platform for diverse biological research applications [5,6]. Despite these strengths, wide-scale adoption of the technology faces a distinct challenge; the availability of robust and accessible quantitative analysis tools that remove reliance on coarse shape metrics and/or qualitative interpretation.

The development of these tools is hindered by the unique nature of AFM data. The community is frequently challenged by proprietary file formats instituted by the manufacturers, which limit the use of external analysis workflows. Furthermore, AFM data present specific processing hurdles that cannot be solved using tools designed for nonsurface techniques. AFM requires specialized preprocessing to correct for specific artifacts such as row misalignment, scanner drift, and tip convolution, and feature extraction must account for surface topography rather than intensity-based contrast common in optical methods. While a host of powerful AFM analysis tools exist, e.g., Gwyddion [7], NanoLocz [8], and WSxM [9], there is not a clear standardized workflow for automated, high-throughput polymer analysis that can generate detailed statistics on molecular conformation.

This is in contrast to the broader bioimaging community who have seen a shift toward automated workflows for batched quantitative analysis and open-source software. Bioimaging encompasses a diverse array of microscopy approaches, each producing vastly different data types. While manufacturers often provide proprietary analysis software, the community repeatedly returns to open-source, accessible software such as Fiji [10], CellProfiler [11], RELION [12], and Napari [13], all of which provide flexible, modular environments for basic preprocessing to the most complex analyses. The open-source and modular nature of these tools also enables researchers to adapt and build upon existing workflows for specialized samples and to develop new analysis methods. Machine-learning-based tools such as DeepImageJ [14] have added a new dimension to image analysis by providing convenient integration of advanced segmentation and classification into workflows. The persistent use of open-source, community-driven workflows, instead of bespoke tools by the bioimaging community, reflects its motivation for reproducible and accessible software.

In response to the need for a unified, accessible, and quantitative tool for AFM image analysis, we present TopoStats v2.0.0, an enhanced successor to TopoStats [15] that supports more complex sample types and enables richer biomolecular characterization. TopoStats v1.0.0 provided 20 metrics describing object or molecule geometry, whereas TopoStats v2.0.0 provides a total of 68 metrics across several additional categories. TopoStats now provides an automated pipeline comprising 5 key stages (Fig. 1): (a) image loading via AFMReader, (b) preprocessing, (c) deep-learning-augmented object detection, (d) morphological analysis for objects of interest, and (e) molecular tracing and analysis; and was developed in accordance with the Findable, Accessible, Interoperable, and Reusable (FAIR) for Research Software (FAIR4RS) principles [16].

**Fig. 1.**
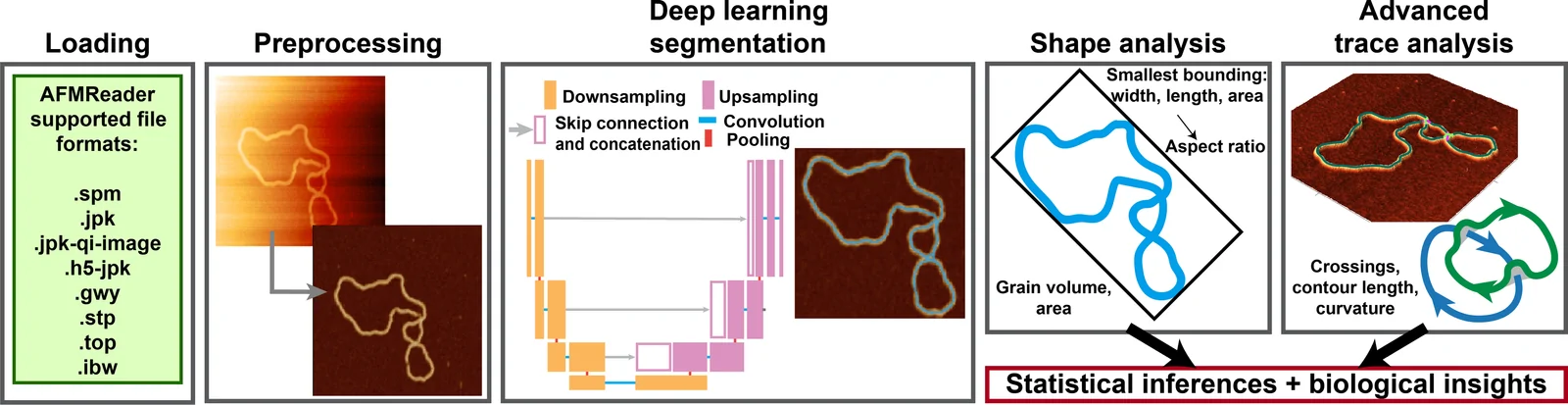
TopoStats processing and quantification pipeline. TopoStats takes raw atomic force microscopy (AFM) data, preprocesses, and segments objects of interest through a combination of thresholding and deep learning segmentation. An advanced DNA trace analysis pipeline then quantifies the complex conformations of molecules, which are able to be used for statistical analysis.

To highlight the full capabilities of this workflow, we use DNA as an exemplar biomolecular system with tunable length and topological complexity. We show that we can segment individual molecules and trace their paths, providing quantitative measurements of their length, flexibility, and degree of self-interaction, driven by sequence or topological changes. DNA topology is fundamental to numerous biological processes and interactions; for example, supercoiling and DNA secondary structures are known to regulate transcriptional activity [17–21], as well as protein binding events [22–24]. The conformational variability of DNA often makes measurements of these effects difficult, as they cannot be described as a single structural class. Single-molecule AFM measurements have been shown to be an effective way to image and describe the structure of DNA, with polymeric analysis showing indicators of metrics such as contour and persistence length [1,25,26]. However, these measurements often require manual intervention or are based on in-house developed code and are therefore difficult to compare between laboratories and samples. Here, we demonstrate TopoStats as a powerful tool for the quantification of biomolecules between datasets and across a range of samples, showing how the number of crossings and curvature vary for DNA in different topological states. We then explore how these metrics are altered when the DNA contains an AT-rich tract compared to a telomeric repeat insert. Overall, TopoStats provides a robust, open-source pipeline for the quantification of AFM data across a wide range of biomolecular samples.

## Methods

All code for TopoStats, including its development history is publicly available on GitHub (https://github.com/AFM-SPM/TopoStats). Documentation, including instructions for installing and running the software, is hosted via its documentation website (https://afm-spm.github.io/TopoStats/main/index.html). The main methods and algorithms implemented in TopoStats are detailed in the this section, and all configurable parameters are listed and described in Table S1.

### Image loading

AFM images are loaded using our AFMReader software (GitHub: https://github.com/AFM-SPM/AFMReader; documentation website: https://afm-spm.github.io/AFMReader/). AFMReader incorporates a mixture of community-built packages and in-house decoding scripts for the loading of its various supported file formats (Fig. S1). Images in binary formats are loaded using Python and decoded according to their formats. AFMReader loads images and extracts the image data and the pixel size in nanometers, which allows for the conversion between pixels to real-world length. Occasionally with some formats, the pixel size is not stated but, instead, must be calculated from available metadata for the size and resolution of the image. Once these quantities have been obtained, they are stored in a unified format and returned to the calling script, able to be used interchangeably regardless of file type.

### Image preprocessing

Raw AFM images undergo a series of preprocessing stages to remove imaging artifacts ahead of downstream analyses. First, scan-line artifacts are corrected by subtracting the median height of each scan row to eliminate row-wise *z*-offsets and align the background to a uniform zero height. Tilt removal is achieved by fitting and subtracting a plane based on the median heights of each row and column. A second-order polynomial is then fitted to the row medians and subtracted to correct parabolic curvature. Finally, a fit of the form z=a×x×y is applied to remove saddle-shaped distortions. After these corrections, the overall image median is subtracted to restore the background to zero height. When many foreground objects are present, flattening becomes less accurate because median-based background fitting fails. To address this, the image is reflattened while masking out foreground regions identified through height-based thresholding. Height masks are binary arrays distinguishing background and foreground pixels. Three thresholding options are available: Otsu, standard deviation (SD), and absolute. Otsu thresholding automatically determines a threshold for bimodal height data, scaled by a user-defined factor. SD thresholding sets the threshold as the mean height plus a user-defined multiple of the SD. Absolute thresholding uses a fixed, user-specified height value in nanometers. Finally, to reduce high-gain noise in the image, a Gaussian filter is applied with user-configurable strength, set to 1 pixel by default.

### Object segmentation

#### Traditional segmentation

Objects of interest are identified using height-based masking, as in the flattening stage. The image is thresholded according to 1 of 3 user-configurable methods, Otsu, SD, or absolute, each independently adjustable from the flattening thresholds. The resulting binary object mask can optionally exclude any objects touching the image edges, as these cannot be measured reliably because of incomplete visibility. Objects that are either too large or too small can also be removed on the basis of user-defined area thresholds (nm^2^), with mask pixels outside these limits set to zero.

#### Deep learning segmentation

After initial object detection, a deep learning model can optionally be applied to refine segmentation and improve accuracy. Each detected object is cropped from the image, and a user-defined padding is added around the bounding box to adjust the relative scale of the object. The crop is then converted into a square by expanding the shorter dimension of the bounding box, while ensuring that it remains fully within the image boundaries. This process produces square image patches centered on each object of interest, suitable for deep-learning-based resegmentation. The deep learning model is loaded from a user-specified path, and each identified object is resegmented using a normalized image crop. For each crop, pixel height values are first clipped to user-defined lower and upper normalization boundaries. The lower boundary is then subtracted, and the result is divided by the difference between the upper and lower boundaries, rescaling all values to the range 0 to 1. This normalization ensures input consistency required for deep learning inference. Finally, each crop is resized to match the input resolution expected by the loaded model. The model then generates a refined segmentation for each object. The batch and channel dimensions are removed, and the floating-point prediction is converted to a binary mask using a user-defined sensitivity threshold (default 0.5), corresponding to the probability at which the model is more confident that a pixel belongs to the foreground than the background. The resulting segmentation mask is then resized to match the original crop dimensions. Before reintegration into the overall object mask, the segmentation result is optionally checked for any newly introduced regions that are disconnected from the original threshold-based segmentation. This step prevents erroneous inclusion of nearby, unrelated objects. A new blank binary mask is then populated with the validated segmentations and used in all subsequent analysis steps.

### Close end joining

Objects of interest often contain low regions of single-stranded DNA that can cause the binary masks to be split up into multiple, disconnected masks. We implemented a simple algorithm to try to identify and connect the mask ends, crossing the gaps to produce a full mask again. This process starts with producing a skeleton of the object mask. This skeletonization process is identical to the main skeletonization process used later on in the pipeline, where the mask is smoothed, skeletonized using height-based weighting and pruned of spurious branches. After a skeleton has been produced, “end points” and “junction points” are identified via convolution. “End points” are points that are at the ends of skeleton branches and have only one-pixel neighbor. “Junction points” are points that reside in the intersections of multiple branches and have more than 2 neighbors. Each point is compared to all other points; the distances between them are calculated and, if within a user-configurable distance, flagged as potential connections. Many points may be within range of each other, so groups of candidates are created, and then once all groups have been constructed, they are individually considered. If there are 2 end points and 0 “junction points” in the group, then these are simply connected to each other. If there are 4 end points and 0 “junction points” in the group, then an algorithm searches for any hard-connected “end points”—ones that are already connected by the skeleton. The aim of this is to identify 2 “end points” that are not directly connected via skeleton to any others in the group, as these are likely to be the entry and exit points for the group. An algorithm follows the skeleton from each “end point” and stores the “end points” that are connected to each other. If there are exactly 2 “end points” that are not connected, then processing can continue; otherwise, the case is discarded. Assuming that 2 “end points” are not connected via skeleton, labeled points A and D, 2 pathfinding options are produced using Dijkstra’s algorithm with a cost based on the inverse of the height map to produce best routes: ABCD and ACBD. The shortest route is then taken, as it is assumed that it is less likely for the separated fragment to be markedly rotated. If there are exactly one “end point” and one “junction point” in the group, then the “end point” and “junction point” are connected. Dijkstra’s algorithm, using a cost map of the inverse height of the image, is used to produce a path between points to be connected, then a measure of the average width of the mask is made, and the single-pixel path is dilated until it matches the average width of the mask, connecting the points together.

### Obtaining statistical descriptors for objects of interest

The spatial morphology of each segmented object is quantified using a suite of geometric and height-based metrics (Fig. S2). When applicable, measurements in pixel units are converted to nanometers by multiplying by the pixel-to-nanometer scaling factor the appropriate number of times (once for length, twice for area, and thrice for volume). Each object is assigned a unique identifier (grain_number), corresponding to the order of indexing within the full-image segmentation mask.

The object’s center coordinates are computed as the mean of all pixel coordinates within its mask. Radius statistics are derived from the set of Euclidean distances between the center and each edge pixel, while height statistics are calculated from all pixel height values in the mask. The object area is defined as the number of pixels within the mask, and the volume is defined as the sum of their height values. The Cartesian bounding box area corresponds to the area of the smallest axis-aligned bounding box containing the object. In contrast, the smallest bounding length, width, and area refer to the minimum dimensions of the tightest bounding box independent of image orientation. To compute this, a convex hull is generated using the Graham scan algorithm, and bounding boxes are iteratively constructed by rotating each hull simplex to the *x* axis via a rotation matrix. From these, the smallest bounding area and dimensions are identified. Minimum and maximum Feret diameters are obtained similarly by iterating over each hull simplex and measuring the distances between opposing hull edges. All calculated statistics are stored and exported in CSV file format for further analyses (Tables S2 and S3).

### Strand tracing

Strand tracing consists of 3 main stages: skeletonization for identifying branches, crossing analysis for determining the route strands take through crossings, and vectorization that reconstructs complete molecular traces from this information. A comprehensive description of the tracing steps can be found in the software documentation: https://afm-spm.github.io/TopoStats/main/advanced/disordered_tracing/ and in detail below.

#### Skeletonization

Before skeletonization, the object mask (Fig. S3A) is smoothed to reduce jagged edges that can produce spurious branches not representative of the true structure (Fig. S3B). Two smoothing methods are available: dilation and Gaussian. Dilation performs a user-configurable number of morphological dilations, thickening and smoothing the mask edges, while Gaussian smoothing applies a Gaussian filter, followed by thresholding at 0.5 to restore a binary mask. If both are enabled, they are applied in parallel, and the method that alters the fewest pixels relative to the original mask is retained. To preserve internal structure, holes are tracked during smoothing; any hole removed can optionally be reinserted if its area lies within user-defined thresholds. Skeletonization is performed using a modified Zhang algorithm [27] that incorporates height information (Fig. S3C). In each iteration, the pixels marked for removal are ordered by their height values, and only the proportion of pixels below a user-specified height percentile is removed. For example, a threshold of 0.4 removes only the lowest 40% of pixels by height, biasing the skeleton toward higher regions of the image. Branches, crossings, and end points are identified by convolving the binary skeleton with a 3 × 3 kernel of ones. This produces a map in which each skeleton pixel is assigned a value corresponding to the number of connected neighbors. Pixels with 2 neighbors are classified as branches, those with 3 or more as junctions and those with one as end points. Spurious branches are then removed in a pruning step. Junction pixels (those with ≥3 neighbors) are temporarily removed, isolating the branches, which are then analyzed individually. Branches may be pruned if they are shorter than a user-defined minimum length or if their height statistics (minimum, median, or midpoint height) fall below a configurable threshold (Fig. S3D). There is also an option to set whether to only prune branches by height if they contain an end point.

Skeleton statistics are then computed using the Skan library [28], which indexes each branch, its connected branches, and its classification (e.g., end-point-to-end-point, junction-to-end-point, junction-to-junction, or loop). Branch lengths, measured in pixels by Skan, are converted to nanometers and summed to obtain total branch length. The number of end points and junctions is also recorded. The average strand width is calculated by performing a distance transform on the object mask, sampling the resulting distances along the skeleton, and averaging them. All measurements are stored as part of the object’s statistics and exported to CSV.

#### Crossing analysis

Crossing analysis identifies and characterizes regions where strands overlap within the skeletonized structure. The process involves 4 main steps: detection of crossing regions, correction of fragmented crossings, branch pairing, and determination of strand stacking order. Crossing regions are first identified by locating junctions, points where 3 or more branches of the skeleton meet, and merging those that lie within a user-defined distance. For DNA data, a distance of approximately 6 to 8 nm is typically appropriate, corresponding to roughly twice the apparent DNA width of 2 nm. The algorithm iterates over each branch extending from a junction and traverses it for the specified distance; if another junction is encountered within this range, the connecting pixels are flagged to mark them as part of the same crossing region. In some cases, a true crossing involving an even number of strands is erroneously split into multiple junctions containing an odd number of branches. To correct this, crossing regions that exhibit odd branch counts and lie within a user-specified distance are merged. The number of emanating branches for each junction is determined by counting adjacent pixels connected to the skeleton.

Each crossing region is analyzed to determine how branches enter and exit. For every branch connected to a crossing, a user-defined segment length is traced and averaged to form a representative vector from the crossing center toward the branch. Branches are then paired on the basis of the alignment of their vectors. Two pairing strategies are available: (a) iterative pairing of the most closely aligned branches until none remain or (b) exhaustive pairing evaluation, selecting the combination with the lowest total alignment error. In cases with an odd number of branches, the user can specify whether pairing should proceed or be skipped. To reduce the impact of variations between individual skeletons on the tracing output, a trace averaging step is applied. Two morphological dilations are performed on the connected branches, and the result of the first is subtracted from the second, generating 2 parallel traces approximately one pixel apart. These, along with the original trace, are averaged to yield a smoother and more representative strand path. The stacking order of each branch pair, indicating which strand passes over the other, is then determined. Each branch is traversed over a configurable distance, and its height profile is extracted. From the middle 40% of this segment, the maximum height is identified, and the full width at half maximum (FWHM) is computed. Restricting analysis to the central portion of the branch reduces interference from nearby crossings. Branches with larger FWHMs are interpreted as lying above those with smaller values. A confidence value is calculated as the ratio between the minimum and maximum FWHMs within each pair. At this stage, statistics including the number of crossings, mean and minimum confidence of crossings, and stacking order are recorded and stored for subsequent analysis. Further detail on the verification of crossing assignments is available in previously published work [29]

#### Vectorization

The skeleton is reanalyzed using connected component analysis after temporarily removing pixels belonging to crossing regions. Each branch is linked to its corresponding stacking-order information from the crossing analysis, assigning a pseudo-height that reflects its relative position within the molecule. Tracing begins at an end point, if present, or with the first branch index otherwise, but never within a crossing segment. The branch is followed, and its coordinates are added to the overall trace. When a segment ends, the adjacent segment with the highest stacking order is followed next, allowing the trace to progressively build. Each completed segment is removed from the mask to prevent retracing. The process continues until no further segments remain, with disconnected regions treated as separate molecules, resulting in vectorized traces for all objects (Fig. S3E). A simplified pseudo-trace is also generated, assigning integer pseudo-heights (0, 1, 2, …) from the stacking order and subsampling the points to fewer than 100 to reduce its complexity. This simplified trace is passed to Topoly [30] to determine the object’s topological classification and Jones polynomial, which are recorded in the object statistics. Finally, the molecule’s writhe is derived from the vectorized trace by computing cross products between adjacent branch vectors aligned along the trace direction. Overpassing and underpassing crossings are denoted by “+” and “−”, with multiple crossings within a region enclosed in parentheses. The resulting writhe string is stored in a CSV file of detailed molecular statistics (Table S3).

### Trace smoothing

Trace smoothing is applied to the vectorized skeleton traces using either spline fitting or rolling average smoothing. In the spline method, multiple B-splines are generated and averaged to produce a smoother and more representative path. The number of splines is determined by a user-defined interval, which specifies how frequently coordinates from the trace are sampled to construct each spline. For example, if the interval is set to 3 nm and trace coordinates are spaced 1 nm apart, 3 splines are generated, each offset by one coordinate. Splines are computed using the splprep function from SciPy [31], with configurable parameters for smoothing strength (separately for linear and circular traces) and spline degree. The sampled splines are averaged to yield the final smoothed trace. Alternatively, rolling average smoothing applies a moving window of user-defined width to locally average neighboring points along the vectorized trace. Following smoothing, the trace is resampled to enforce uniform point spacing. This is achieved by iterating through the coordinates, calculating distances between successive points, and interpolating or skipping points as needed to maintain consistent spacing. The resulting trace provides a smooth and evenly sampled representation of the original vectorized skeleton, suitable for downstream analysis.

### .topostats file format

The introduction of the .topostats file format (a custom Hierarchical Data Format version 5 [HDF5]-based structure) provides modularity and flexibility for users who wish to access or repurpose outputs from different stages of the TopoStats pipeline. Users can extract intermediate data such as flattened images, segmentation masks, or molecular traces for further processing, visualization, or integration with external analytical tools. These outputs can be used directly within Python or saved as standard image files or NumPy arrays for import into common image analysis software such as ImageJ. Our TopoStats GitHub repository provides notebooks that illustrate how to interact with .topostats files, guiding users through the extraction of relevant data and demonstrating how custom functions can be developed for bespoke analysis workflows, thereby giving users more autonomy in how they use the software.

### AFM sample preparation and imaging

Eight nanograms of supercoiled, nicked, AT insert or telomeric insert DNA was immobilized on a freshly cleaved mica disk in 30 μl of immobilization buffer (3 mM NiCl_2_ and 20 mM Hepes [pH 7.4]) for 5 min. The mica was then washed 4 times with the same buffer, and a further 30 μl was added for imaging. All AFM measurements were performed in liquid following a previously published protocol [32]. All experiments were carried out in PeakForce Tapping imaging mode on a FastScan Dimension XR AFM system (Bruker) using FastScan D (Bruker) probes. The PeakForce amplitude was set to 6 nm, the PeakForce Tapping frequency was set to 8 kHz, and the PeakForce set points were in the range of 7 to 15 mV, corresponding to peak forces of <70 pN. All images were taken at 0.5 nm per pixel at line rates of ∼3 to 5 Hz.

### Dataset image processing

The acquired images were then processed using TopoStats using the config file provided with the dataset. Deep learning segmentation was performed with a U-Net previously trained and demonstrated in Holmes *et al.* [29]. Briefly, training was performed on 76 hand-labeled cropped AFM images (upscaled to 512 × 512 pixels), which underwent random augmentations of up to a 30% scale increase, up to a 30% translation, integer rotations of 90°, and horizontal or vertical flipping. The model was trained with a learning rate of 0.001, a batch size of 5, and 120 steps per epoch and trained for 100 epochs. The U-Net architecture was constructed as a 5-layer encoder–decoder network with skip connections with specific parameters (see Holmes *et al.* [29]). Using a test dataset of 15 images, the resultant trained model obtained a dice score of 0.82 and intersection over union score of 0.70 on pixel probabilities.

Processing on this dataset resulted in a total of *n* = 57 supercoiled, *n* = 57 nicked, *n* = 70 telomeric insert, *n* = 52 AT-rich insert molecules being retained for this analysis. No molecules were excluded on the basis of the crossing confidence.

### 3AT and telomeric repeat insert sequences

The 3AT insert sequence is as follows: 5′ atcctatatatatatatatatatatatatatgcgaactcatatatatatatatatatatatatatggtgccgtcatatatatatatatatatatatatat 3′; The Telomeric DNA insert sequence is as follows: 5′ ccctaaccctaaccctaaccctaaccctaaccctaaccctaaccctaaccctaaccctaaccctaaccctaa 3′.

## Results

### Standardized AFM file loading with the AFMReader Python package

Loading and extracting data from AFM image files are notoriously difficult because of competing manufacturer file types, standards, and insufficient documentation. While existing analysis software [7,9] can handle these files, they are limited in their ability to interface with modern Python libraries, which restricts downstream analysis. There is some community effort to provide tools to make these formats more accessible, with libraries such as pySPM [33] and igor2 [34]; however, they are not unified, and their outputs have to be handled on a case-by-case basis. We developed AFMReader [35], a Python package that unifies support for multiple AFM file formats within a single framework, using both existing, community-made libraries and bespoke loading apparatus. This is designed with interoperable open-source code that is thoroughly tested, incorporating comprehensive documentation and community involvement. AFMReader currently supports the most widely used file types from major AFM manufacturers and, using the HDF5 file structure, provides a unified output to enable the development of processing scripts and analysis software that are agnostic to instrument or file type (Table S5). It functions both as an independent Python package and as an integrated module within TopoStats, extending the software’s applicability across all supported AFM file formats.

### Automated preprocessing of AFM images to enable quantitative analysis

Once loaded, raw AFM images need to be preprocessed prior to downstream analysis to remove AFM-specific imaging artifacts [36]. TopoStats facilitates a series of configurable processing steps (Fig. S1) including tilt correction for correcting image slope, row alignment to remove scan-line artifacts, and an algorithm to remove scars—long, thin horizontal streaks in images due to the AFM tip erroneously skipping across the surface. Finally, the image data can be shifted so the background has a height of 0, allowing for reliable comparison of molecular heights between objects in multiple images.

During slope and row correction, issues can occur when surface features interfere with background estimation. In such cases, the algorithm may incorrectly interpret object regions as part of the background, causing the slope removal to perform poorly and leading to rows containing objects being overcorrected. This results in those rows appearing darker in the processed image and the object heights being underestimated (Fig. S4A). To mitigate this effect, we introduced 2 improvements. First, the quantile used to estimate the background level for each row can now be user-configured, allowing finer control over how the algorithm defines the background height. Second, the preprocessing pipeline has been divided into 2 sequential stages. The initial stage performs standard image flattening and correction procedures to produce a preliminary image. The secondary stage then identifies nonbackground regions using a user-defined automatic thresholding method, masks these regions, and performs a second round of flattening restricted to background pixels. This 2-step approach yields a more reliable and uniform image flattening, even in datasets containing a high density of surface features (Fig. S4B). As well as improving the batch processing capability of TopoStats, this advance in flattening enables a higher sample throughput by use of higher surface concentrations of biomolecules, a factor that is typically limiting in single-molecule approaches.

### Automated segmentation and feature extraction from AFM images

#### Classical segmentation methods

To extract information on molecular conformation, objects of interest must first be identified through segmentation to isolate the regions intended for measurement. In AFM topographs, objects of interest typically differ in height from the background, making height-thresholding a reasonable and effective approach for segmentation (Fig. S5A). We implemented 3 commonly used methods for height thresholding: Otsu, SD-based, and absolute thresholding. The Otsu thresholding method estimates the optimal cutoff value for a bimodal height distribution, generating a mask of all pixels above this threshold. This approach is particularly effective when objects of interest exhibit similar, well-defined heights relative to the background. The absolute thresholding method allows direct specification of the threshold in nanometers, making it useful when the approximate height of the target structures is known or a precise cutoff is desired. The SD method defines the threshold as the mean height of the grains plus a user-configurable multiple of the SD, providing flexibility for cases where object heights are unknown or vary widely according to a normal distribution.

#### Segmentation clean-up steps

After an initial segmentation mask is generated (Fig. S5B), cleanup is required to ensure that only objects of interest are retained in the segmentation mask for analysis. Objects that intersect the edge of the image cannot be reliably measured since an indeterminate amount of the object lies outside the image bounds, and so these objects are optionally removed (Fig. S5C, blue). Furthermore, objects of interest may clump together and overlap, or there may be contamination in the image that could interfere with analysis. These can be removed using configurable area thresholds, removing objects that are smaller or larger than the expected object size (Fig. S5D, purple). The result is a mask that ideally depicts just the objects intended for downstream analysis and quantification.

#### Deep learning methods for improved segmentation

The quality of downstream analysis is directly tied to the accuracy of sample segmentation, making the generation of an accurate mask essential. Conventional height thresholds, while effective for simple, well-defined samples, often struggle when applied to heterogeneous data where objects of interest vary in height, overlap, or display only subtle topographical contrast with the background. The increasing use of deep learning models [37,38] has driven huge advances in segmentation tools [39–41] that, in some cases, exceed the capabilities of traditional approaches. Having demonstrated the power of deep learning models to accurately segment complex biological species [22,29], we elected to integrate deep learning into the segmentation functionality of TopoStats. Using tools such as LabelStudio, users can label images, highlight specific features of interest, and use this to train a deep learning model. Once trained, users can add their bespoke U-Net model easily within TopoStats by linking it to the standard configuration file where it will be used as part of the segmentation workflow. The implementation of deep learning models in TopoStats provides users with the tools to further refine their image analysis and enhance object segmentation for their unique sample. The example provided in this work is applicable to small (<3 kb) plasmids; however, alternative models would be required for different sample types, which appear visually different. An example of segmentation improvements from our small plasmid model is shown in Fig. 2. Each region of interest is cropped, padded by a configurable amount, normalized, and then run through the segmentation model to produce a mask that preserves fine structural details of objects of interest that traditional methods often obscure. This U-Net mask aligns more closely to the center of the molecule backbone, which is vital for accurate downstream tracing. Figure S6 demonstrates the increased accuracy of the tracing pipeline for molecules segmented with a U-Net compared to classical thresholding. The differences are especially clear in regions where strands pass close to one another—the classical threshold often joins these strands or creates “false” crossings, where the U-Net thresholding stays true to the path of the molecule.

**Fig. 2.**
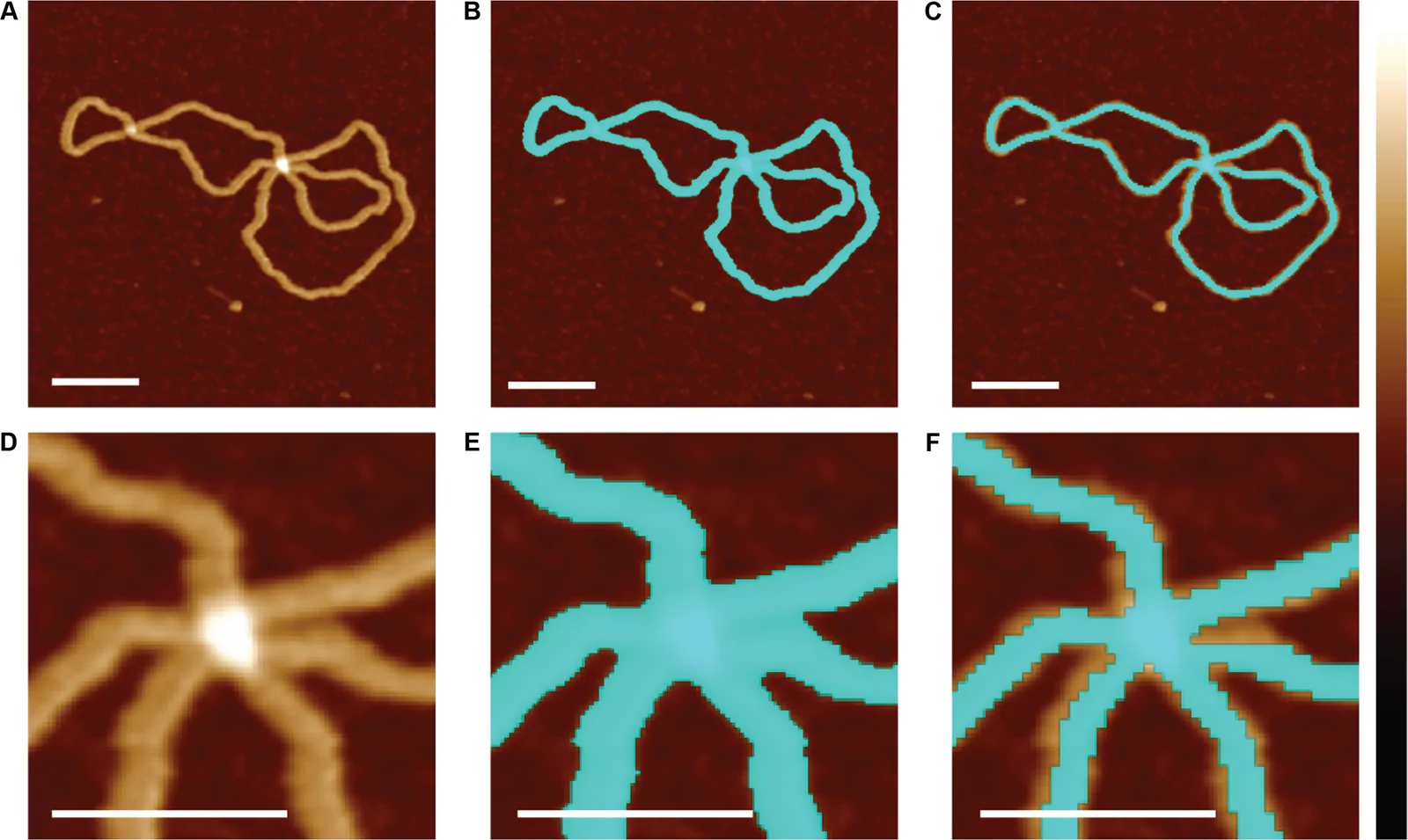
Deep learning to refine object segmentation. Atomic force microscopy (AFM) image of a DNA molecule (A). Traditional height-thresholded mask of the molecule (B). U-Net segmentation of the molecule enables better masking (C). Zoom-ins of a particularly difficult to segment crossing of the catenane highlighting the improvement due to the U-Net segmentation (D to F). Scale bars, 50 nm. Height scale, −3 to +4 nm.

Once objects have been successfully segmented, their corresponding height map pixels are used to calculate quantitative morphological statistics. We implemented a suite of scripts to generate a comprehensive set of statistics, including spatial and geometric information, such as the position, aspect ratio, Feret diameters, and bounding areas, and height-based information including height and volume (Fig. S2). These statistics are output into a human-readable CSV file indexed by image, folder name, and object number for easy identification and comparison between datasets.

### An advanced skeletonization pipeline for identification of molecular backbones

For filamentous samples such as DNA, fibrils, or polymer chains, global morphological statistics can fail to fully describe conformational differences between objects. In these cases, it is often more informative to determine the molecular contour length or width and determine whether individual molecules adopt a linear or closed-loop conformation. While other strand tracing pipelines allow for calculation of these metrics [15,26], they cannot accurately identify and trace self-interaction events, where 2 strands cross over each other. These events are important to resolve to understand how polymers are interlinked and how often they cross one another. Correctly tracing strands as they pass over or under one another is essential for accurately describing molecular topology [29] and enables the separation of overlapping or intersecting molecules. These metrics are a useful indicator of conformational state, for example, when distinguishing relaxed and supercoiled DNA, and can be used to quantify the effects of conformation-altering drugs [42]. We developed a sophisticated tracing pipeline that harnesses the unique topographical information available from AFM images to produce reliable traces of molecular backbones, including at intersecting points.

#### Optimizing segmentation masks for skeletonization

The first step is to generate a representative skeleton—a single-pixel-wide mask that accurately delineates the central axis of the segmentation mask (Fig. 3A and B). As skeletonization is applied directly to the mask, it is often necessary to preprocess masks, for example, by smoothing edges or filling small holes, to minimize the formation or erroneous skeleton branches. To support this, we implemented 2 preprocessing options: (a) Gaussian smoothing followed by rebinarizing using Otsu thresholding and (b) binary dilation. Gaussian smoothing is effective for reducing noise in the mask while maintaining the shape of the grains. The second method, binary dilation, performs morphological dilation to smooth and connect adjacent regions of the mask, which can help close small gaps and strengthen thin regions of a mask. Both methods yield a smoother mask edge and reduced instances of spurious branches in the skeletonized trace. In some instances, the trace can break as it passes through low regions of the molecule (for example, a region of single-stranded DNA within a plasmid). To avoid unnecessary grain removal during data cleanup due to these incomplete traces, we implemented an end joining module that detects and joins nearby end points (Fig. S7). This, in turn, allows gaps in segmentation masks to be retrospectively filled and the affected grain to reenter the analysis workflow.

**Fig. 3.**
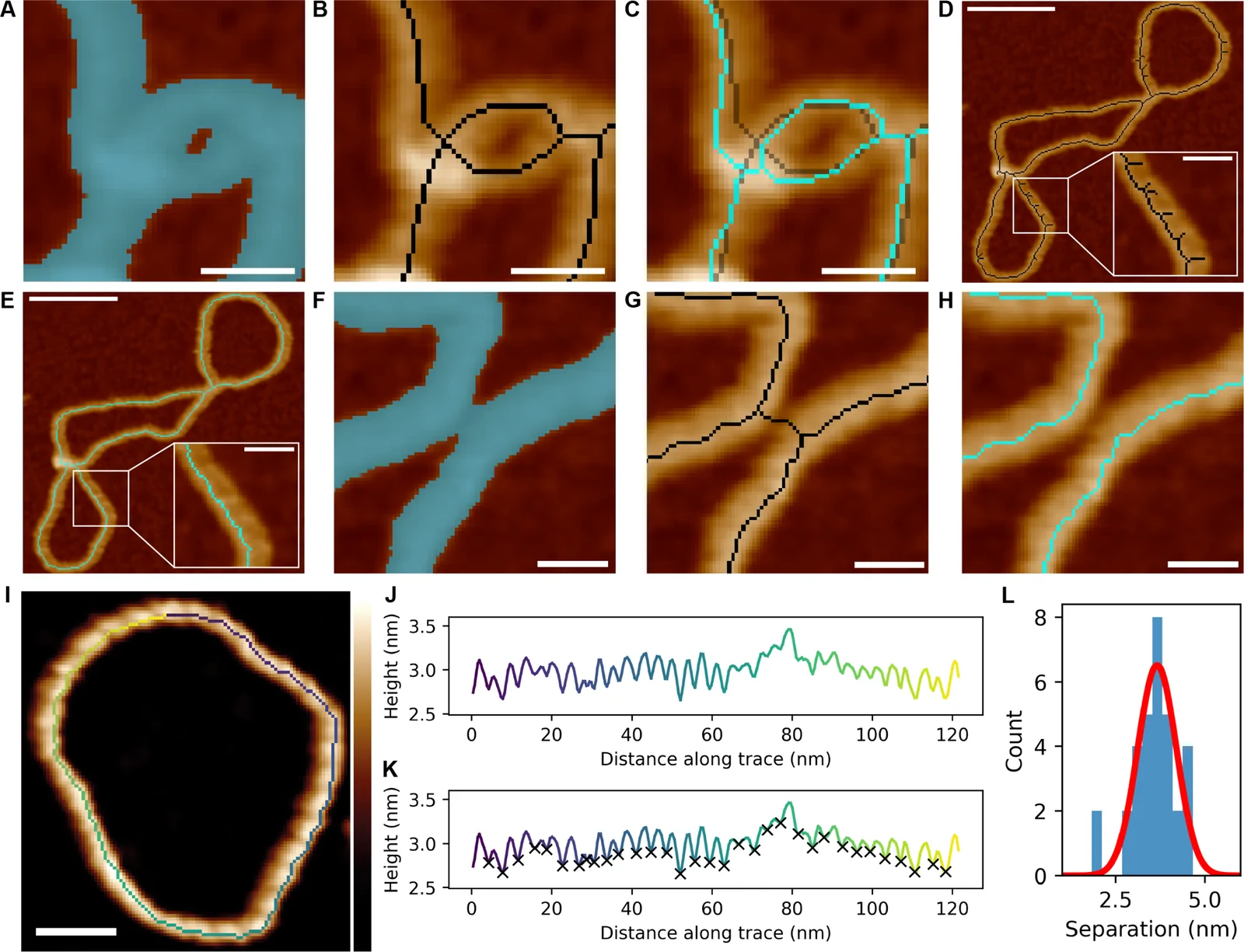
Advanced skeletonization analysis removes errors allowing full plasmid traces to be obtained. Height biasing applied during the skeletonization of a single pICOz molecule mask (A) pulls unrepresentative skeletons (B) toward higher and more representative regions of DNA strands (C). Skeleton pruning of a single pICOz trace (D) removes erroneous trace branches (E). Skeleton pruning based on height of branches between 2 close, but separate, pICOz strands (F) removes the erroneous connecting branch (G and H). Accurate skeletonization of minicircle DNA (I) yields a height trace (J), which allows the detection of the major grooves location (K). Measurement of the major groove spacing, with a Gaussian fit in red (L). Scale bars, 10 nm. Height scale, −3 to +4 nm (A to H) and −0.5 to 4 nm (I).

#### Height-biased skeletonization optimizes molecular traces

Following mask preprocessing, a single-pixel representation of the object mask can be obtained using a traditional skeletonization method such as the Zhang algorithm [27]. However, in AFM images, tip convolution introduces positional uncertainty, meaning that the molecular backbone is best represented by the highest regions of polymeric strands. Traditional binary morphological skeletonization methods do not account for the underlying height information produced by AFM, often resulting in skeletons that deviate from the true molecular backbone (Fig. 3B). We therefore developed a customized version of Zhang’s skeletonization algorithm that biases the skeleton toward higher regions of strands. In each iteration, the algorithm identifies pixels eligible for deletion but removes only a user-defined fraction of them, preferentially retaining those corresponding to local height maxima. This approach guides the skeleton to remain centered on the highest pixels throughout the thinning process, producing a final skeleton that more closely follows the true molecular backbone, thus providing a more reliable representation of the molecule (Fig. 3C). Despite mask preprocessing, erroneous branches can still occur. To address this, we include a pruning algorithm that removes spurious branches based on their length and the underlying AFM height information (Fig. 3D and E). This addition is motivated by the observation that erroneous branches often do not lie over regions of the molecule of interest but, instead, extend into background areas or bridge 2 regions across a background gap (Fig. 3F to H). Branches essential for maintaining skeleton continuity are preserved to prevent fragmentation of a single molecule into separate traces. Full and accurate skeletonization enables a host of advanced analysis features including determining the periodicity of surface features [43]. Tracing the backbone of the molecule (Fig. 3I and J) enables us to measure subtle height changes along the molecule with a high degree of accuracy, such as the major groove spacing around a full DNA minicircle, 3.7 ± 0.6 nm (Fig. 3K and L). This value is in good agreement with the accepted value of 3.4 nm for B-DNA within experimental uncertainty and other AFM measurements of helical repeat [3,44].

### Complex crossing analysis for topological resolution of intersecting polymer strands

Beyond height tracing, accurate skeletonization enables a pipeline that can determine the topology of DNA plasmids and more complex structures by identifying over- and underlying strands. Once a skeleton has been obtained and erroneous branches have been removed, crossing points can be identified as pixels in the skeleton that are connected to more than 2 neighboring pixels, corresponding to locations where 2 strands intersect (Fig. 4A).

**Fig. 4.**
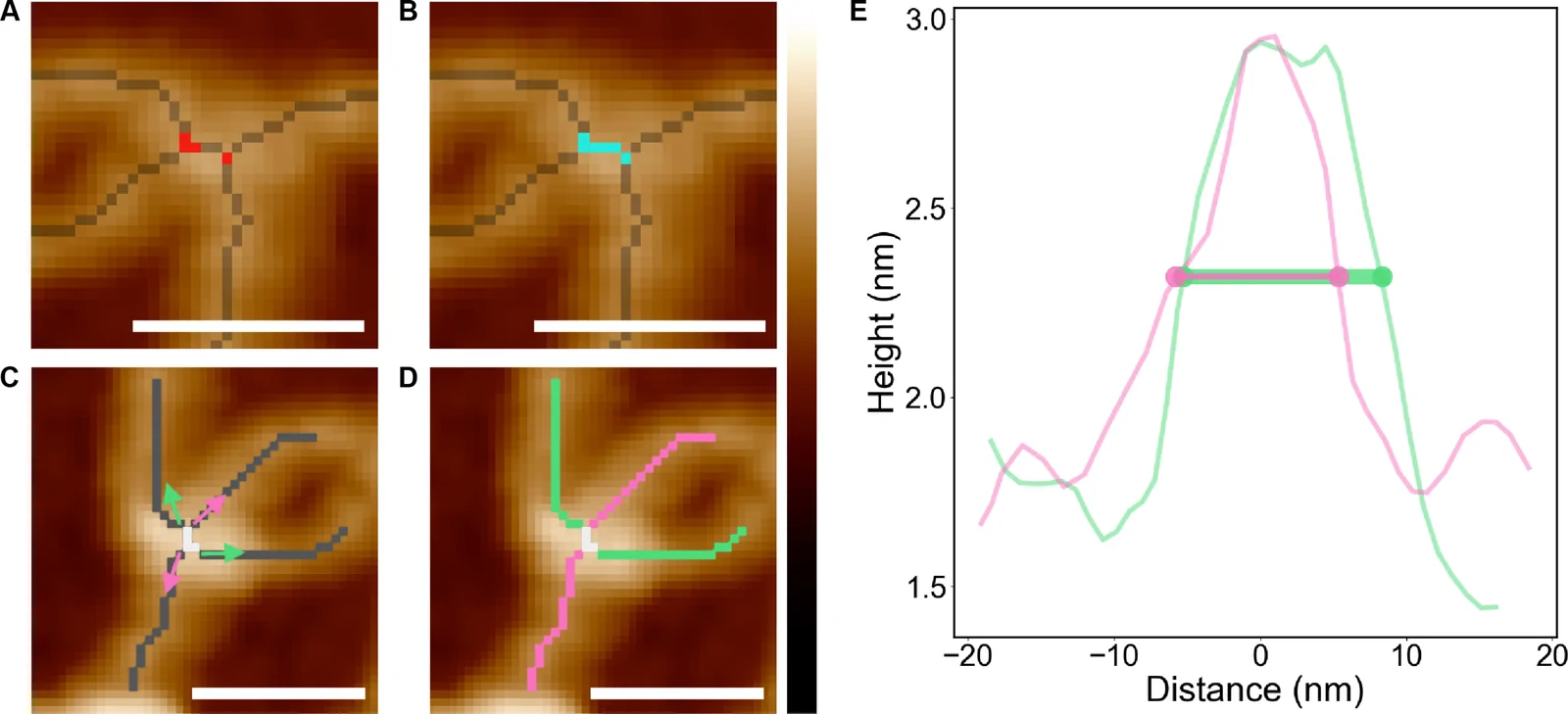
Detailed junction analysis enables tracing DNA through crossings. Merging of 2 separately identified junctions (A) into a combined junction (B), enabling consideration of all 4 strands as part of one crossing. Vectors calculated from the strands (C) are used to connect similarly oriented strands to determine DNA paths through the crossings (D). (E) Consideration of the heights of each strand pair allows determining which strand pair lays over the other, by calculating which has a largest full width at half maximum height (E). Scale bars, 40 nm. Height scale, −3 to +4 nm.

#### Pairing branches at crossing points

Having identified these crossing points, the next step is to pair the emanating branches to achieve a continuous trace of the entire molecule. In some cases, what should represent a single intersection point is detected as multiple nearby crossing points. This effect arises from AFM tip convolution, which broadens the contact region between strands, extending the crossing area beyond its true dimensions (Fig. 4A). Before determining how segments connect and which strand passes over or under, crossing points in close proximity are first merged and treated as a single node (Fig. 4B). Once close nodes have been correctly consolidated, each crossing region is examined in turn, and the branches emanating from it are identified. Segment connectivity through each crossing is determined by analyzing the angles formed by the branches emanating from the crossing center. These angles are calculated by representing each branch as a single vector, obtained by following the branch for a user-defined distance and averaging the corresponding point coordinates (Fig. 4C). Here, we use a 20-nm crossing region, below the 45-nm persistence length of DNA [45], to avoid issues with determining the vector of curved strands. These emanating branches are then paired on the basis of their bipartite graph maximal matching vectors [29], according to the closest matching angles, indicating the most likely continuation of the molecule through the crossing (Fig. 4D). In some cases, crossings may contain an odd number of branches. In such instances, the algorithm either performs best-effort pairing, leaving one branch unpaired, or omits pairing entirely, based on user defined configuration parameters informed by sample morphology.

#### Determining the stacking order of branches

The stacking order of overpassing and underpassing strands, i.e., which strand lies on top and which lies below, is determined by analyzing the height profiles of the strands as they pass through each crossing. Overlying and underlying DNA strands exhibit distinct height characteristics: The overlying strand gradually rises before reaching its peak height above the crossing, whereas the underlying strand remains relatively flat before showing a sharp increase as it passes beneath the other. This observation can be quantified by calculating the FWHM of each strand’s height profile. The overpassing strand consistently exhibits a higher FWHM, while the underpassing strand has a lower value (Fig. 4E). Using this FWHM approach for crossing classification can be limited by low-quality, noisy height profiles. Therefore, the difference between the FWHM values is used to assess the confidence in correctly identifying the over- and underpassing strands, with a larger difference indicating greater certainty in the classification. Each molecule is assigned an overall mean confidence that can be used to assess and filter on the basis of the quality of the crossing assignment.

### Reconstruction of continuous DNA traces enables topological identification

Combining tracing and crossing analysis, TopoStats can reconstruct molecules as continuous traces. This is achieved by following connected pixels from a starting branch along the skeleton (Fig. 5A) and through predetermined crossings to create a full, uninterrupted trace of the molecular contour (Fig. 5B and C). In some cases, multiple molecules may be connected within the same grain, for example, if 2 molecules are catenated (Fig. 5D to F) or overlapping (Fig. 5G to I), making it inaccurate to represent them as a single entity. To address this, the algorithm continues tracing unvisited branches and identifies them as separate molecules. This functionality is particularly valuable for tracing complex topological structures or to perform instance segmentation and separate analysis of distinct but overlapping molecules.

**Fig. 5.**
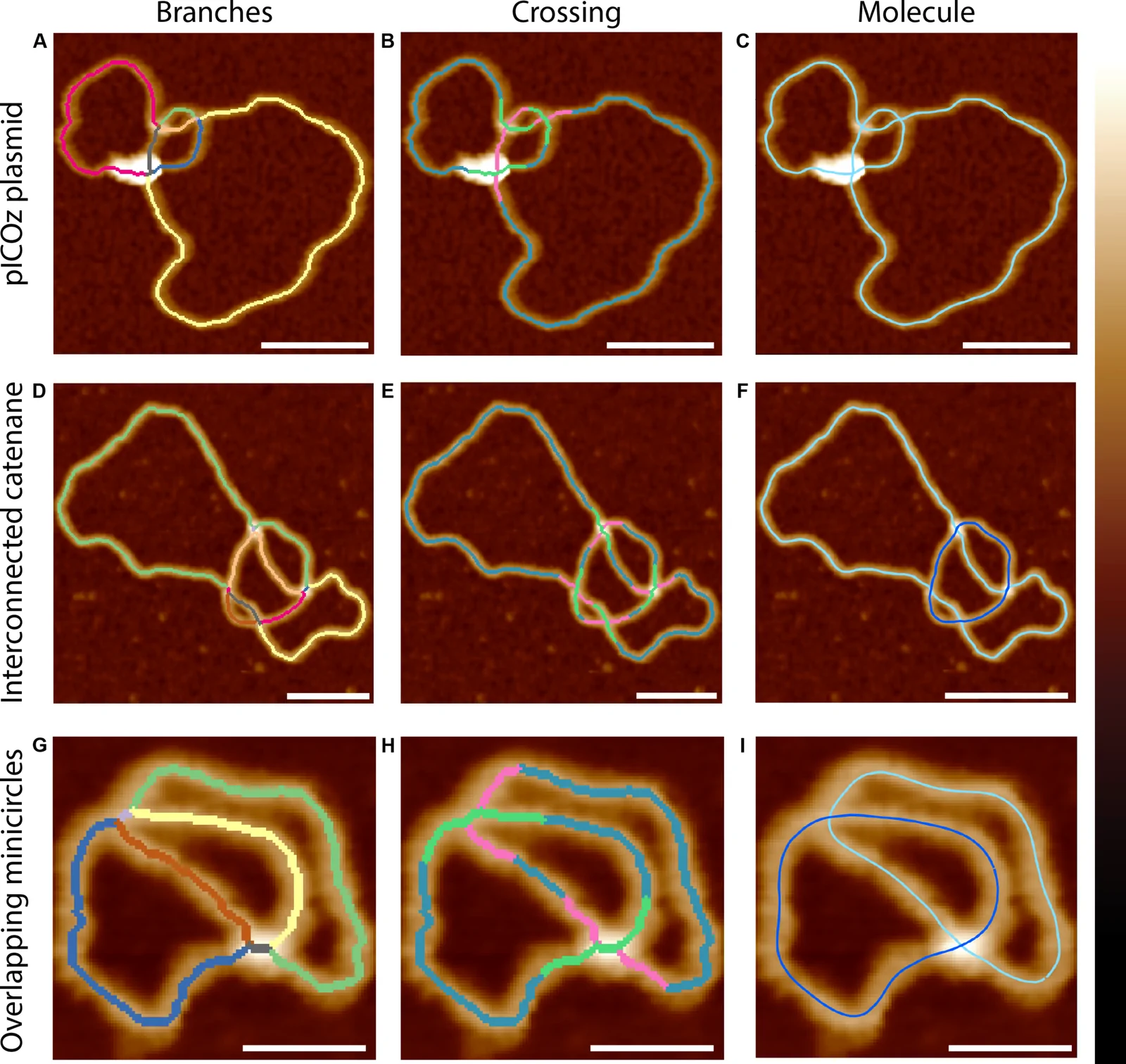
Trace vectorization enables individual molecule identification. Splining and molecule identification from atomic force microscopy (AFM) images of a DNA plasmid exhibiting self-interactions (A to C), an interconnected DNA catenane (D to F), and 2 overlapping DNA minicircles (G to I). Each of the skeleton branches obtained from the tracing are displayed in a different color (A, D, and G). Crossing determination is carried out with overlying strands marked in pink and underlying strands marked in green (B, E, and H). Smoothed trace of the plasmid using the rolling average method (C). The 2 interconnected molecules of the catenane have been automatically identified (small plasmid in dark blue and large plasmid in light blue) (F), and the 2 overlapping minicircles have been identified (each individual molecule spline is highlighted in dark or light blue) (I). Scale bars, 40 nm (A to C), 80 nm (D to F), and 20 nm (G to I). Height scale, −3 to +4 nm.

#### Trace smoothing to reconstruct the molecular backbone

Using pixel-based images for analysis inherently produces traces that are jagged, as each coordinate is constrained to the pixel grid, limiting smoothness to the resolution of the image. To overcome this, spline fitting can be applied, in which a mathematical curve is fitted through the trace points and resampled to generate a smoother trace. While effective in most cases, spline fitting requires careful parameter tuning to match the geometry of each molecule. If these parameters are not optimized, then the spline can deviate from the true molecular path, particularly around tight bends, yielding an unrepresentative trace where the trace “falls off” the molecule and touches the background of the image. To address this limitation, we implemented an alternative smoothing method based on a rolling average that requires only a single, user-defined window size in nanometers (Fig. 5C, F, and I). This method is more reliable than splining, ensuring that traces do not stray from the molecule, and requires less optimization by the user. Following smoothing, trace coordinates may be unevenly distributed with some regions dense in coordinates and others fairly sparse. To address this, we incorporated a resampling step that enforces uniform spacing of trace coordinates, with the rate of sampling able to be set by the user to increase or decrease the number of points as required for further analysis. The implementation of splining provides the foundation for measuring further tracing-based parameters that yield additional information to the grain-based parameters (Fig. S2).

The implementation of splining provides the foundation for calculating additional tracing-based metrics that extend beyond shape descriptors such as aspect ratio. Using the generated spline, we can calculate curvature; this parameter captures changes in molecule shape by measuring how sharply the trace bends locally. By quantifying the variation in curvature, we can determine changes in molecule conformation on the surface and make inferences about the underlying mechanics.

### Accurate tracing metrics show significant differences between molecular topologies

#### Supercoiled plasmids show a significant increase in number of self-crossings compared to nicked plasmids

Supercoiled DNA is known to have a more compact conformation due to superhelical stress that manifests as writhe, wherein 2 strands cross over themselves [44,46]. Conversely, nicked DNA has been shown to have a more open conformation, a consequence of a break in a single strand of the DNA backbone that releases the superhelical tension; nicked plasmids are less prone to self-crossing. We imaged both supercoiled and nicked pICOz (1,277 bp) plasmids (*n* = 57 supercoiled and *n* = 57 nicked) and extracted grain- and trace-based statistics. When comparing the grain-based metric, aspect ratio, we detected no differences between the supercoiled and nicked population (Fig. S8A). For both supercoiled and nicked samples (Fig. 6A and B), the mean contour length measured from the spline was close to the expected plasmid length of 434 nm, with a length of 419 ± 56 and 435 ± 111 nm for supercoiled and nicked, respectively (Fig. 6C).

**Fig. 6.**
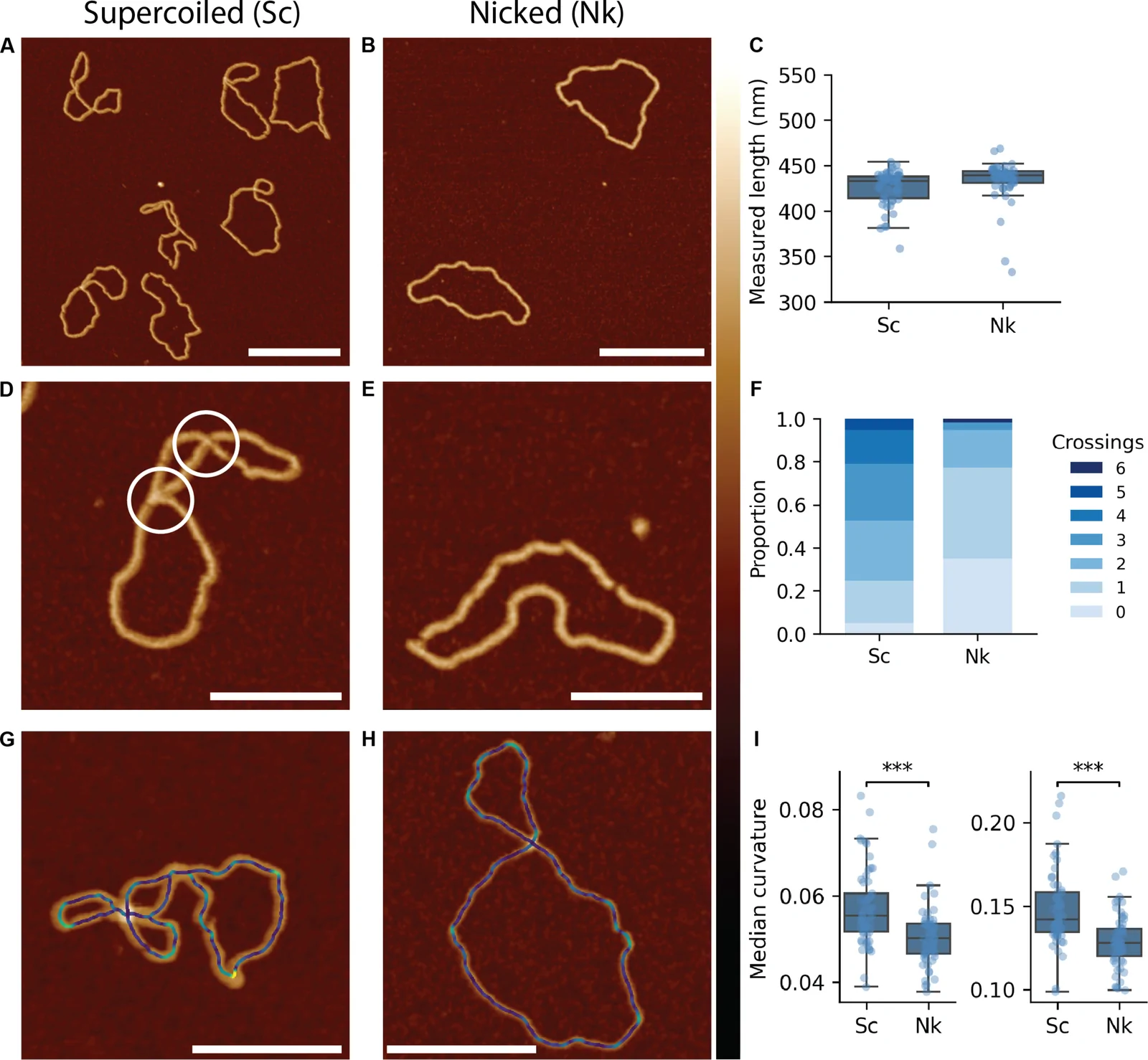
Calculation of crossing numbers allows for quantification of more subtle conformational changes. Representative zoom-out atomic force microscopy (AFM) images of multiple supercoiled (A) and nicked (B) pICOz DNA plasmids. Contour length box plots (C) for supercoiled and nicked datasets (expected pICOz length: 433 nm). Representative zoom-ins of a supercoiled (D) and a nicked (E) pICOz plasmid. Stacked bar charts displaying the proportion of numbers of crossings in a plasmid between supercoiled and nicked pICOz plasmid datasets (F). Visualizations of curvature intensity for representative plasmids of both types (G and H). A comparison of the median and 90th percentile of curvature values for both sample types (I). Scale bars, 160 nm (A and B) and 80 nm (D to H). Height scale, −3 to +4 nm. Statistical significance for the median curvature was determined using a Student’s *t* test, and that for 90th percentile of curvature was determined using a Mann–Whitney, nonparametric *t* test. ***P ≤ 0.001.

To demonstrate the importance of each part of the TopoStats pipeline on the quality of the final trace and therefore the contour length measurement, we performed a series of ablation tests on the supercoiled pICOz data (Fig. S9 and Table S6). Performance was quantified using residual scores, calculated as the difference between the measured, and expected contour length value (434 nm). The U-Net (catsnet)-enhanced segmentation (Fig. S9A) outperformed the optimal classical segmentation (residual 2.7 ± 7.4 nm *versus* 3.6 ± 7.5 nm), providing accurate measurements of molecule length. When overmasking (*h* > 0.2 nm), we find an increase in the *N* for the dataset (62 compared to the expected 58), suggesting that spurious areas of the image are being masked. We also find an increase in the residual score for the contour length due to false branches being created by the mask edges. When undermasking (*h* > 1.9 nm), we find a decrease in the *N* (50), suggesting that molecules are being missed because of the high threshold. Contour length residual scores were larger when each element of the TopoStats pipeline was disabled: end joining, 3.5 ± 7.5 nm (Fig. S9B); pruning, 4.7 ± 8.0 nm (Fig. S9C); height biasing, 3.7 ± 7.8 nm (Fig. S9D); trace smoothing, 3.8 ± 7.3 nm (Fig. S9E). These measurements verify that the deep learning segmentation, along with the trace optimization modules, enhances the accuracy of the TopoStats measurement pipeline.

Crossing analysis revealed a significant change in the crossing distributions between the supercoiled and nicked plasmid populations, proving a potent metric for determining conformation and demonstrating the heterogeneity of the plasmids in each group (Fig. 6D and E). The supercoiled population had a mean crossing average of 2.4 ± 1.3 compared to the nicked population, which was significantly lower at 1.0 ± 1.0 (***P = 1.5 × 10^−9^) (Fig. 6F).

#### Curvature analysis shows differences in molecular conformation between supercoiled and nicked plasmids

Understanding how DNA flexibility responds to changes in supercoiling requires metrics that capture local curvature variation along the molecular backbone. Here, the curvature is calculated by a rate of change of direction of the spline, with quick changes in direction measured as high curvature and slow changes in direction as low curvature (Fig. 6G and H). Curvature statistics calculated for supercoiled and nicked plasmids showed that nicked plasmids exhibit a significantly reduced average median curvature (0.050 ± 0.007) and 90th percentile of curvature (0.12 ± 0.02) compared to the supercoiled plasmids (median = 0.057 ± 0.009, ***P = 7.4 × 10^−6^; 90th percentile = 0.15 ± 0.02, ***P = 3.8 × 10^−7^) (Fig. 6I). The reduced median curvature indicates that nicked plasmids adopt a more uniform and smoothly curved conformation and the lower 90th percentile demonstrates a lack of local curvature maxima, consistent with the more open and circular structures observed in the AFM images. Conversely, the curvature statistics of the supercoiled plasmids describe conformations with tighter global and local bends, reflecting their torsionally constrained nature. These results demonstrate the utility of this method for generating accurate quantitative statistics and reliably distinguishing between different DNA topological states at the single-molecule level.

### Changes in sequence-driven plasmid flexibility are described by curvature metrics

These advanced tracing statistics can be further leveraged to explore how changes in DNA sequence impact molecular conformation. Using 2 variants of the pICOz plasmid, pICOz with 12 repeats of the telomeric insert, TTAGGG (pTEL12; Fig. 7A), and pICOz with an AT-rich insert (p3AT; Fig. 7B), we were able to quantify how changing the local GC composition of the plasmids affected its flexibility and shape (*n* = 70 telomeric insert and *n* = 52 AT-rich insert). The 72-bp telomeric insert in the pTEL12 has a GC content of 50%, whereas the AT-rich insert of the p3AT plasmid measures 100 bp and comprises only 14% GC. GC pairs form 3 hydrogen bonds between opposing bases compared to the 2 bonds formed between AT pairs, which contributes to increased structural rigidity of the DNA helix. This increased rigidity due to the high GC content is reported to reduce the ability of the helix to compensate for torsional strain through change in twist [47]. Consequently, when negative supercoiling is introduced into the helix, the GC-rich telomeric region may resist this underwinding. Unable to accommodate increased torsional strain as twist, to minimize the energetic cost within the constrained pTEL12 plasmid, it is plausible that a greater proportion of the torsional strain is partitioned into writhe resulting in an increased number of crossings (3.2 ± 1.6; Fig. 7D and F) and corresponding higher median (0.06 ± 0.01) and 90th percentile curvature (0.16 ± 0.02) (Fig. 7G and I). In contrast, the p3AT plasmid, which contains a greater proportion of AT base pairs in the same region, may be more flexible and therefore able to effectively accommodate changes in torsional strain through change in twist (Fig. 7E and F). The p3AT plasmids exhibit significantly fewer self-crossings (1.8 ± 1.4, ***P = 8 × 10^−6^) and a significantly smoother curvature profile (average median = 0.05 ± 0.01, ***P = 0.0003; average 90th percentile = 0.14 ± 0.02, ***P = 0.0005) compared to the pTEL12 plasmid.

**Fig. 7.**
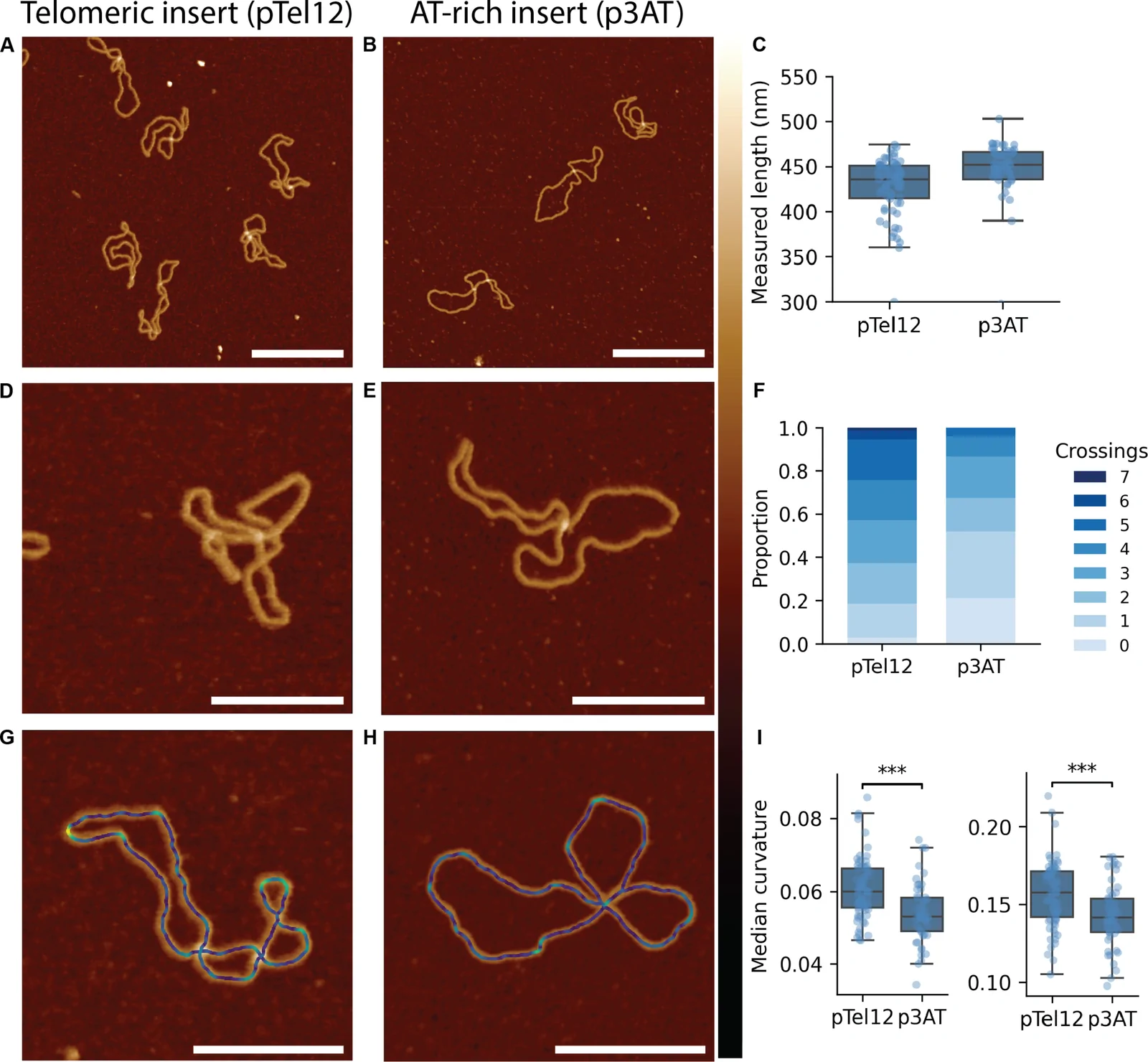
Using TopoStats to measure the effect of DNA sequence on plasmid conformation. Atomic force microscopy (AFM) images of DNA plasmids with telomeric (A) and AT-rich (B) inserts. A comparison of the measured lengths of the 2 plasmid types (C). Representative zoom-ins of the plasmids (D and E). Comparison of the number of self-crossings present in the 2 plasmid types (F). Visualizations of curvature intensity for representative plasmids of both types (G and H). A comparison of the median and 90th percentile of curvature values for both sample types (I). Scale bars, 160 nm (A and B) and 80 nm (D to H). Curvature crops, 80 nm. Height scale, −3 to 4 nm. Statistical significance for the median curvature was determined using a Mann–Whitney, nonparametric *t* test, and that for 90th percentile of curvature was determined using a Student’s *t* test. ***P ≤ 0.001.

Given that both plasmids are of a similar length (pTEL12 = 1,356 bp and p3AT = 1,367 bp; Fig. 7C), they should have the same level of native supercoiling (ΔLk = −8) partitioned between twist and writhe. In the absence of sequence-dependent effects, we would therefore expect both the pTEL12 and p3AT plasmids to have the same measured contour length and global topology and thus adopt similar surface conformations, e.g., the same number of crossings and curvature. However, despite comprising only a small fraction of the total plasmid length (5% for pTEL12 and 7% for p3AT), the change in number of crossings and relative curvature suggest that subtle sequence differences may influence the flexibility of the plasmid [48]. This, in turn, will alter the twist-writhe partitioning and therefore the global conformation. Taken together, these data are consistent with the hypothesis that AFM, combined with advanced and accurate molecular measurements, can detect and quantify sequence-dependent changes in plasmid conformation of a surface. More broadly, this highlights the power of TopoStats for studying structural effects of DNA binding proteins, enzymatic activity, and other complex systems influencing DNA topology.

## Discussion

This upgraded version of TopoStats retains the core functionality of the original TopoStats software including image loading and preprocessing, object segmentation, statistical extraction, and data export but has been expanded to introduce several new functionalities. Through the development and integration of AFMReader, we have removed instrument-specific barriers, enabling loading and analysis of AFM images from multiple platforms with varied file formats. We have enhanced image preprocessing to include scar removal and custom row alignment to ensure proper flattening, which is crucial for obtaining accurate statistics downstream. To further improve object segmentation, we have implemented deep learning segmentation into the TopoStats workflow. By making it possible for user-defined TensorFlow deep learning models to be imported as part of the segmentation pipeline, we can handle even more complex and heterogeneous biomolecular systems.

Our new tracing pipeline refines conventional skeletonization algorithms by leveraging the unique height information in AFM data to accurately align skeletons to molecular backbones, even through complex crossings. This allows the generation of accurate height traces to extract information such as the helical periodicity of a molecule. TopoStats overcomes the typical difficulties with tracing through strand intersections by pairing emanating branches, enabling over- and underpassing strands to be distinguished. We demonstrated the efficacy of the tracing pipeline using DNA as a model system, showing that the workflow is able to accurately trace single DNA molecules and is capable of resolving overlapping molecules resulting from random surface adsorption. This functionality enables the technique to serve as a data-cleaning step, lessening the reliance on meticulous sample preparation to prevent molecular overlap and enabling a greater number of relevant molecules to be retained for analysis. The potential of the tracing pipeline is further enhanced by the addition of crossing and curvature analysis, which provides insight into global and local changes in molecular shape. The contributions of each element of the analysis pipeline is assessed, showing that careful optimization of parameters can lead to accurate measurements of DAN contour length. We have shown that nicked and supercoiled plasmids can be distinguished by quantifying changes in curvature and writhe, and these metrics can be used to probe sequence-dependent changes in plasmid conformation and flexibility. Beyond DNA, these tools will enable users across disciplines to explore diverse sample types. Filamentous structures, such as protein assemblies [49,50], in particular amyloids [51,52], and polymer chains [53], which are structurally similar to DNA, could be passed through the same segmentation and tracing pipelines to extract rich structural data. Careful selection of height thresholds or the use of advanced U-Nets can also allow proteins and other globular structures to be analyzed to obtain morphological metrics [22,54]. We anticipate that future versions of TopoStats will handle force-curve-based data, expanding its capabilities toward force spectroscopy analysis [55,56].

TopoStats represents a substantial step toward making quantitative analysis more accessible to the AFM community, advancing a field that remains comparatively underdeveloped relative to other imaging techniques such as optical microscopy. In developing TopoStats, we placed strong emphasis on software design, usability, and reproducibility, guided by the FAIR4RS principles to ensure that the software is findable, accessible, interoperable, and reusable. Findability is supported through public hosting of the software on GitHub, PyPi, and an online documentation site, making each version easily discoverable, citable, and accessible to the community. Accessibility is enhanced through open-source availability and comprehensive documentation covering every stage of the TopoStats workflow, which is automatically generated, versioned, and published online whenever new functionality is added to ensure up-to-date guidance for both users and developers. Interoperability is achieved through the use of open, standardized data formats (CSV, HDF5, PNG, SVG, and TIFF), enabling results to be easily visualized and integrated with other analytical tools. Reusability is supported by robust software-engineering practices, including automated testing throughout the codebase to ensure reliability and consistent functionality during development, a modular architecture that supports batch processing and multicore parallelization for efficient large-scale analysis, and a unified YAML configuration file that contains all processing parameters. Metadata describing the software version, configuration parameters, and analysis conditions are stored alongside results, ensuring transparency and full reproducibility. Designed with user needs in mind, TopoStats fosters community engagement, allowing users to suggest new file format support, contribute novel analytical modules, and help shape future developments through its open GitHub repository. In doing so, TopoStats lays the groundwork for a more open and collaborative, quantitative AFM research.

Collectively, these developments establish TopoStats as an advance for quantitative AFM image analysis, offering a unified, open, and reproducible platform that greatly expands the analytical scope and accessibility of this technique. The extensive toolbox included in TopoStats enables users to extract detailed structural information from their images bridging the gap between AFM imaging and quantitative molecular analysis.

## Data Availability

All data used in this publication is publicly available on Figshare upon publication at the following link (https://figshare.com/s/cfb49fe81be784e7061a), a full DOI will be provided upon publication. Plasmid sequences used in the study are available on GenBank with the following accession numbers—pICOz_Tel12: PZ472946 and pICOz_3AT: PZ472947. All code developed in this publication is publicly available on the main GitHub branch of TopoStats (https://github.com/AFM-SPM/TopoStats) and through PyPI through “pip install topostats”.
