# Supplementary material for: Deep-Learning-Enhanced TopoStats for the Automated Quantification of DNA and Complex Biomolecular Structures
Source: Comput Struct Biotechnol J. 2026 Sep 18;35(1):0227. doi: 10.34133/csbj.0227 (PMC13738353; doi:10.34133/csbj.0227)
Supplement: Supplementary file 1 [file csbj.0227.f1.zip › TopoStats2_ SI_Resubmission_Final.docx]

# Supplementary Information for “Deep Learning-Enhanced TopoStats for the Automated Quantification of DNA and Complex Biomolecular Structures”

Sylvia Whittle^1✝^, Tobias A. Firth^1,2✝^, Max C. Gamill^1^, Laura Wiggins^1^, Neil Shephard^3^, Toby Allwood^1^, Daniel Brady^3^, Thomas E. Catley^1^*, Alice L. B. Pyne^1^*

^1^School of Chemical, Materials and Biological Engineering, University of Sheffield, Sheffield, UK.

^2^School of Medicine and Population Health, University of Sheffield, Sheffield, UK.

^3^Department of Computer Science, University of Sheffield, Sheffield, UK.

^✝^Authors contributed equally.

*To whom correspondence should be addressed. Email: t.catley@sheffield.ac.uk, a.l.pyne@sheffield.ac.uk

| **Section** | **Parameter** | **Default Value** | **Description** |
| --- | --- | --- | --- |
| **General** |  |  | General options |
|  | base_dir | ./ | Directory containing the data to process |
|  | output_dir | **./**output | Directory to output results to |
|  | cores | 2 | Number of CPU cores to utilise |
|  | file_ext | .spm | File type of the data files to search for and load |
| **Loading** |  |  | Options pertaining to loading AFM data |
|  | channel | Height | Image channel to load from the data file |
| **Filter** |  |  | Options pertaining to flattening & preprocessing |
|  | row_alignment_quantile | 0.5 | Quantile to assume the background value is at. Lower values may produce better flattening in images with more data above the surface |
|  | threshold_method | std_dev | Method to use for thresholding objects from the background |
|  | otsu_threshold_multiplier | 1.0 | Multiplier to apply to the calculated Otsu threshold |
|  | threshold_std_dev | 1.0 | Number of standard deviations from the mean to use as the height threshold |
|  | threshold_absolute | 1.0 | Absolute height threshold in nanometres |
|  | gaussian_size | 1.0 | Amount of pixels of gaussian smoothing to apply to the flattened image |
|  | remove_scars/run | false | Whether to interpolate over scars in the image |
| **Grains** |  |  | Options pertaining to identifying objects of interest |
|  | grain_crop_padding | 1 | Number of pixels to pad the grain’s bounding box by |
|  | threshold_method | std_dev | Method to use for thresholding objects from the background |
|  | otsu_threshold_multiplier | 1.0 | Multiplier to apply to the calculated Otsu threshold |
|  | threshold_std_dev | 1.0 | Number of standard deviations from the mean to use as the height threshold |
|  | threshold_absolute | 1.0 | Absolute height threshold in nanometres |
|  | area_thresholds | 300, 3000 | Lesser and greater area thresholds in nm^2 below / above which grains are removed |
|  | remove_edge_intersecting_grains | true | Whether to remove grains that intersect the image bounds |
| **unet_config** |  |  | Options pertaining to the re-segmentation of identified objects of interest using a user-provided, pre-trained TensorFlow deep-learning model |
|  | model_path | null | System path to a trained TensorFlow model |
|  | upper_norm_bound | 5.0 | Pre-segmentation upper normalisation bound |
|  | lower_norm_bound | -1.0 | Pre-segmentation lower normalisation bound |
|  | remove_disconnected_grains | false | Whether to remove regions in predicted masks that do not intersect the original mask |
|  | confidence | 0.5 | The confidence threshold for the binarisation of the prediction |
| **disordered_tracing** |  |  | Options pertaining to skeletonisation |
|  | mask_smoothing_params/gaussian_sigma | 2 | Amount of gaussian smoothing to use when smoothing a mask before skeletonisation |
|  | mask_smoothing_params/dilation_iterations | 2 | Number of morphological dilation processes to run when smoothing a mask before skeletonisation |
|  | mask_smoothing_params/holearea_min_max | [0, null] | The minimum and maximum size in nm^2 of holes in the mask pre-smoothing to re-add after smoothing |
|  | skeletonisation_params/height_bias | 0.6 | Percentage of pixels to remove from the candidate pixels, ordered by height. Lower value results in stronger biasing towards high regions, higher values approach a Zhang-style skeletonisation |
|  | pruning_params/max_length | 10 | Maximum length in nm to remove a branch that does not connect to another branch |
|  | pruning_params/height_threshold | null | The height below which to remove branches |
|  | pruning_params/method_values | mid | The method to obtain a branch’s height for determining if it should be removed |
|  | pruning_params/method_outlier | mean_abs | The method for determining outlier heights of branches |
|  | pruning_params/only_height_prune_endpoints | false | Whether to only apply height based branch removal to branches that do not connect to other branches |
| **nodestats** |  |  | Options pertaining to crossing analysis |
|  | node_joining_length | 7.0 | Distance in nm to connect nearby crossing points |
|  | node_extend_dist | 14.0 | Distance in nm to join nearby odd-branched crossings |
|  | branch_pairing_length | 20.0 | Distance in nm to follow a branch to collect height data for crossing analysis |
|  | pair_odd_branches | false | Whether to try to pair branches of crossings with odd-numbers of branches |
| **splining** |  |  | Options pertaining to smoothing & splining |
|  | method | rolling_window | Whether to use splining or rolling window averaging for trace smoothing |
|  | rolling_window_size | 20.0e-9 | Size of the rolling average window, in metres |
|  | rolling_window_resampling | true | Whether to resample rolling-window averaged points to ensure they are equally spaced along the trace |
|  | rolling_window_resample_regular_spatial_interval | 0.5e-9 | The distance in metres that resampled traces’ points should be spaced |
|  | spline_step_size | 7.0e-9 | The sampling rate in metres of the spline(s) |
|  | spline_linear_smoothing | 5.0 | The smoothing for splines that do not connect to themselves to form a loop |
|  | spline_circular_smoothing | 5.0 | The smoothing for splines that do connect to themselves to form a loop |
|  | spline_degree | 3 | The degree of the spline polynomial |
| **plotting** |  |  | Options pertaining to plotting of results |
|  | savefig_format | null | What format to save images in, either PNG, SVG, PDF |
|  | savefig_dpi | 100 | The DPI for each saved output image |
|  | grain_crop_size_nm | -1 | The size of the crops in nm for grains when the “grains” setting for image_set is used. Null results in tight bounding boxes |
|  | image_set | core | The verbosity of output images. Core just produces the minimum. Add more modules to get detailed visual output for them |
|  | zrange | Null, null | The minimum and maximum values in the colour scale for output images. Null results in using the minimum and maximum for the image |

**Supplementary Table 1 - TopoStats’ basic configuration options.** The most commonly used and generally applicable configuration options of TopoStats2, provided in a .yaml file.

| Parameter | Description |
| --- | --- |
| image | Filename of the image |
| image_size_x_m | Width of the image in metres |
| image_size_y_m | Height of the image in metres |
| image_area_m2 | Area of the image in metres squared |
| image_size_x_px | Width of the image in pixels |
| image_size_y_px | Height of the image in pixels |
| image_area_px2 | Area of the image in pixels squared |
| grains_number | Number of objects of interest found |
| grains_per_m2 | Number of objects of interest per metre squared |
| rms_roughness | Root mean square roughness of the image |

**Supplementary Table 2 - Global image statistics.** Statistics produced by TopoStats2 pertaining to each whole AFM image, allowing quantification and comparison of global metrics.

| Parameter | Description |
| --- | --- |
| image | Filename of the image |
| basename | Parent folder (dataset) name |
| grain_number | Index of the object of interest |
| centre_x | Centre x coordinate of the object (m) |
| centre_y | Centre y coordinate of the object (m) |
| radius_min | Minimum radius of the object (m) |
| radius_max | Maximum radius of the object (m) |
| radius_mean | Mean radius of the object (m) |
| radius_median | Median radius of the object (m) |
| height_min | Minimum height of the object (m) |
| height_max | Maximum height of the object (m) |
| height_mean | Mean height of the object (m) |
| height_median | Median height of the object (m) |
| volume | Volume of the object (m3) |
| area | Area of the object (m2) |
| area_cartesian_bbox | Area of the bounding box for the object (m2) |
| smallest_bounding_width | Length of the shorter axis of the smallest possible rectangular bounding box not aligned to cartesian coordinates (m) |
| smallest_bounding_length | Length of the longer axis of the smallest possible rectangular bounding box not aligned to cartesian coordinates (m) |
| smallest_bounding_area | Area of the smallest bounding box not aligned to cartesian coordinates (m2) |
| aspect_ratio | Ratio of the axes of the smallest possible bounding box not aligned to cartesian coordinates. |
| max_feret | Maximum feret diameter (m) |
| min_feret | Minimum feret diameter (m) |
| grain_endpoints | Number of strand end-points in the object |
| grain_junctions | Number of junction pixels in the object’s skeleton |
| total_branch_length | The sum of the lengths of all branches of the object’s skeleton |
| grain_width_mean | The mean width of the strands in the object |
| num_mols | The number of individual molecules contained in the object |
| writhe_string | A string of the under/over status of all the object’s crossing regions. |
| average_end_to_end_distance | The mean distance between the object’s strand end-points |
| total_contour_length | The total length along all smoothed ordered traces for all identified molecules |

**Supplementary Table 3 - Object statistics.** Statistics produced by TopoStats2 pertaining to each individual object in each AFM image, with a mixture of general geometric properties and more specific DNA-focused measurements.

| Parameter | Description |
| --- | --- |
| image | Filename of the image |
| basename | Parent folder (dataset) name |
| grain_number | Index of the object of interest |
| molecule_number | Index of the molecule within the object |
| circular | Whether the molecule contains a self-connection. |
| topology | Topological classification of the object. Ie, linear, 0_1, 3_1, etc. |
| contour_length | Length of the molecule (m) |
| end_to_end_distance | Euclidean distance between linear molecules’ endpoints |

**Supplementary Table 4 - Detailed molecule statistics.** Extra statistical information about sub-grains and molecules identified by TopoStats2, where multiple molecules or objects intersect and should be handled as a single whole object, but are identifiable as different structures.

| **File format name** | **Description** |
| --- | --- |
| **Supported file formats** |  |
| .spm | Bruker primary format |
| .jpk | Bruker acquired format |
| .jpk-qi-image | Bruker QI imaging mode |
| .h5-jpk | Bruker open acquired format |
| .gwy | Gwyddion file format |
| .ibw | WaveMetric format |
| .stp | WSXM AFM format |
| .top | .stp format variant |
| .topostats | TopoStats HDF5-encoded output |
| .asd (partial) | High speed AFM format |
| **Unsupported file formats** |  |
| .nhf | Nanosurf format |
| .aris | Imaris Oxford Instrument format |
| .tiff | Common image format |

**Supplementary Table 5 .** AFM file formats currently supported in TopoStats2 via AFMReader.

|  | **Contour Length (nm) (Mean ± STD)** | **Residual Score, x10^-8^ (Mean ± STD)** |
| --- | --- | --- |
| Optimal Classical | 408 ± 79 | 3.6 ± 7.5 |
| Catsnet (Unet Segmentation) | 412 ± 76 | 2.7 ± 7.4 |
| Low Height Threshold (0.2 nm) | 385 ± 136 | 8.4 ± 11.7 |
| High Height Threshold (1.9 nm) | 349 ± 107 | 8.7 ± 10.5 |
| Optimal Classical, No End Joining | 407 ± 79 | 3.5 ± 7.5 |
| Optimal Classical, No Pruning | 395 ± 85 | 4.7 ± 8.0 |
| Optimal Classical, No Height Biasing | 401 ± 80 | 3.7 ± 7.8 |
| Optimal Classical, No Trace Smoothing | 420 ± 81 | 3.8 ± 7.3 |
| Optimal Classical, everything off | 400 ± 87 | 4.7 ± 8.1 |

**Supplementary Table 6 .** Mean and standard deviation values for the contour length and residuals for the ablation studies.

**Supplementary Figure 1. Image filtering and flattening process in TopoStats2.** Raw image **(A)**, which subsequently undergoes row alignment **(B)**, tilt removal **(C)**, and quadratic flattening **(D)**. Finally, the median height of the image background pixels are set to zero **(E),** and a user-defined Gaussian blur is applied **(F)**. Scale bar = 100 nm.

**Supplementary Figure 2: Basic grain shape can be quantified using morphological metrics.** A visualisation of the various morphological metrics that are calculated by TopoStats2 for each object of interest, and subsequently output in the results file.

**Supplementary Figure 3. Schematic showing the process grain handling in TopoStats.** First, the grain is segmented using either classical or deep-learning methods **(A)**, then smoothed to remove jagged edges which lead to spurious branches (**B**). This segmentation mask is then skeletonised using a modified Zhang algorithm (**C**), which can be pruned based on a user-defined length (**D**). Finally, the skeleton is passed through the crossing analysis algorithms and vectorisation to produce an “ordered” trace (**E**).

**Supplementary Figure 4. Image flattening artefacts present in TopoStats v1.0 are able to be remedied in TopoStats v2.0.** AFM image **(A)** of a DNA plasmid processed using TopoStats v1.0 method, where a flattening artefact manifests as a thick dark band across the image, erroneously lowering the height of the affected rows. The same AFM image **(B)** processed using TopoStats v2.0 method, where the flattening artefact has been remedied with use of the “row_alignment_quantile” setting being set to 0.1, resulting in no dark band, nor erroneous lowering of rows. Height scale: -3 to 4 nm. Scale bars: 20 nm.

**Supplementary Figure 5. Height masking enables identification of objects of interest.** AFM image **(A)** of multiple DNA plasmids. Initial height-based binary mask **(B)** identifying objects of interest and isolating them from the background. Removal of edge-intersecting objects marked in cyan **(C)**, and objects that are too small or too large marked in magenta **(D)**. Height scale: -3 to 4 nm. Scale bars: 20 nm.

**Supplementary Figure 6. Comparison of classical and U-Net segmentation and downstream tracing.** Example images showing segmentation through the optimised classical pipeline and through the Catsnet U-Net pipeline. Differences in tracing quality are highlighted in the inset boxes.

**Supplementary Figure 7: Process of end-joining to reconnect broken skeletons.**

AFM image of a pICOz plasmid with two height dips **(A)** resulting in a discontiguous mask and skeleton **(B)**. End-points are identified **(C)**, and connected via a constructed skeleton following the highest path between the endpoints and subsequent dilation to produce a filling of appropriate width, resulting in a complete mask, **(D)**.

**Supplementary Figure 8: Basic morphological measurements fail to distinguish supercoiled and nicked pICOz datasets.** Comparison of supercoiled & nicked pICOz **(A)** and telomeric insert and AT rich insert **(B)** DNA plasmid datasets’ aspect ratios. Statistical significance for the aspect ratio of supercoiled and nicked plasmids was determined using a Mann-Whitney, non parametric t-test, and for the telomeric insert and AT rich inserts, a Student’s t-test. Data was tested for normality using a Shapiro-Wilk test.

**Supplementary Figure 9: Ablation study to determine the contribution of each part of the TopoStats pipeline to the final output.** Changes in the segmentation thresholds and method (**A**), end-joining algorithm (**B**), pruning (**C**), height biasing (**D**), and trace smoothing (**E**) are tested and their impact assessed by measuring the resulting contour length (**F**). Accuracy is measured by taking the residual of the measurement from the ground truth contour length (434 nm) (**G**).
